# Correction: Comparison of short-term clinical outcomes between low-dose prasugrel and clopidogrel as part of triple antithrombotic therapy in patients requiring oral anticoagulant therapy and percutaneous coronary intervention

**DOI:** 10.1371/journal.pone.0293937

**Published:** 2023-10-31

**Authors:** Hideki Kitahara, Kazuya Tateishi, Yuki Shiko, Yusuke Inaba, Yoshio Kobayashi, Takahiro Inoue

The fourth author’s name is spelled incorrectly. The correct name is: Yosuke Inaba

## References

[1] Kitahara H, Tateishi K, Shiko Y, Inaba Y, Kobayashi Y, Inoue T (2022) Comparison of short-term clinical outcomes between low-dose prasugrel and clopidogrel as part of triple antithrombotic therapy in patients requiring oral anticoagulant therapy and percutaneous coronary intervention. PloS ONE, 17(7): e0272140. 10.1371/journal.pone.0272140 35901007 PMC9333269

